# Cystatin C Deficiency Promotes Epidermal Dysplasia in K14-HPV16 Transgenic Mice

**DOI:** 10.1371/journal.pone.0013973

**Published:** 2010-11-15

**Authors:** Weifang Yu, Jian Liu, Michael A. Shi, Jianan Wang, Meixiang Xiang, Shiro Kitamoto, Bing Wang, Galina K. Sukhova, George F. Murphy, Gabriela Orasanu, Anders Grubb, Guo-Ping Shi

**Affiliations:** 1 Department of Medicine, Brigham and Women's Hospital and Harvard Medical School, Boston, Massachusetts, United States of America; 2 Department of Cardiology, The Second Affiliated Hospital, College of Medicine, Zhejiang University, Hangzhou, China; 3 Department of Life Sciences, School of Biotechnology and Food Engineering, Hefei University of Technology, Hefei, China; 4 Department of Pathology, Brigham and Women's Hospital and Harvard Medical School, Boston, Massachusetts, United States of America; 5 Department of Clinical Chemistry, University Hospital, Lund, Sweden; Universidade de Sao Paulo, Brazil

## Abstract

**Background:**

Cysteine protease cathepsins are important in extracellular matrix protein degradation, cell apoptosis, and angiogenesis. Mice lacking cathepsins are protected from tumor progression in several animal models, suggesting that the regulation of cathepsin activities controls the growth of various malignant tumors.

**Methods and Results:**

We tested the role of cathepsins using a mouse model of multistage epithelial carcinogenesis, in which the human keratin-14 promoter/enhancer drove the expression of human papillomavirus type 16 (HPV16) early region E6/E7 transgenes. During the progression of premalignant dysplasia, we observed increased expression of cysteine protease cathepsin S, but concomitantly reduced expression of cathepsin endogenous inhibitor cystatin C in the skin tissue extract. Absence of cystatin C in these transgenic mice resulted in more progression of dysplasia to carcinoma *in situ* on the face, ear, chest, and tail. Chest and ear skin extract real time PCR and immunoblot analysis, mouse serum sample ELISA, tissue immunohistological analysis, and tissue extract-mediated *in vitro* elastinolysis and collagenolysis assays demonstrated that cystatin C deficiency significantly increased cathepsin expression and activity. In skin from both the chest and ear, we found that the absence of cystatin C reduced epithelial cell apoptosis but increased proliferation. From the same tissue preparations, we detected significantly higher levels of pro-angiogenic laminin 5-derived γ2 peptides and concurrently increased neovascularization in cystatin C-deficient mice, compared to those from wild-type control mice.

**Conclusion:**

Enhanced cathepsin expression and activity in cystatin C-deficient mice contributed to the progression of dysplasia by altering premalignant tissue epithelial proliferation, apoptosis, and neovascularization.

## Introduction

Cysteine protease cathepsins are secretory lysosomal proteases that mediate both intracellular and extracellular protein degradation. Besides degrading endocytosed unwanted proteins, cathepsins are critical in degrading extracellular matrix collagen, laminin, fibronectin, and elastin, thus playing essential roles in tumor growth [1], [2]. Secreted and cell membrane–bound forms of tumor cathepsins may relate to local cellular behavior in certain cancers [3], [4]. Studies have described increases in cathepsin levels for most invasive cancer types [5]. In cancers, the best-studied cathepsins include cathepsins B (CatB), L (CatL), and S (CatS). In an immunohistological study using samples from human colon cancer patients, CatB was overexpressed and correlated strongly with angiogenesis and regional lymph node metastasis [6]. CatB inhibition in glioblastoma cells via RNA interference (RNAi) resulted in impaired invasion, reduced cell–cell interaction with endothelial cells (EC), disruption of capillary network formation *in vitro* and *in vivo*, and inhibition of established glioma tumor growth and invasion in intracranial tumors *in vivo* [7], [8]. RNAi-mediated CatB inhibition also reduced meningioma cell migration and invasion and led to significant regression of pre-established orthotopic tumors [9]. In an ischemic hindlimb model, CatL-deficient mice showed reduced neovascularization. CatL-deficient EC were less able to support the vascularization at the site of ischemia than were wild-type EC [10]. Transfection of an anti-CatL single-chain variable fragment in human melanoma cells inhibited the tumorigenic and metastatic properties completely, therefore producing less angiogenic and smaller tumors [11]. Similar observations were made in a RIP-Tag2 transgenic mouse pancreatic islet cell carcinoma model, in which the rat insulin II promoter (RIP) drove expression of the SV40 large T-antigen (Tag). These tumors also had increased cathepsin expression, which associated with angiogenic vasculature along the invasive front. Reduction in the activity of cathepsins using the pharmacologic inhibitor JPM, or deficiency of CatS, impaired angiogenic switch in progenitor lesions, affecting angiogenesis, tumor growth, vascularity, and invasiveness [12], [13].

Cystatins are endogenous inhibitors of cysteinyl cathepsins. Cathepsin to cystatin ratio increases in most tumor types, compared to normal tissue, particularly for advanced cancers [14]–[17]. Cystatin C, the most important, abundant, and widely expressed cathepsin inhibitor in humans and animals, decreased with concomitant increase of CatB in high-grade gliomas [15]. Benign ovarian cancers had significantly higher cystatin C levels than did malignant tumors [17]; in prostate cancers, cystatin C levels also decreased at late stages [18]. Nearly 50% of cancers show decreased expression of cystatin C, and perhaps of other cystatins [19]. However, tumor cystatin levels vary widely. In prostate cancers, cystatin C levels remained high at early stages [18]. During colorectal cancer progression, cystatin C remained unchanged between stages, whereas CatB levels remained high at any stage [20]. Serum cystatin C levels are significantly higher in patients with ovarian cancers than in benign ovarian tumors or in healthy women, though serum CatB levels are not different [21]. Similar findings are observed in patients with head and neck squamous-cell carcinoma; serum cystatin C levels in these patients are higher than those from control groups [22]. These observations suggest that the relative ratio of cystatin C to cathepsins in tumor tissues *in situ*, and not solely the proteases or inhibitors or those in the circulation, are essential in regulating tumor growth.

One approach to directly evaluate the effect of cystatin C on tumor initiation and evolution uses animal models with either increased or decreased expression of this inhibitor. More than 10 years have passed since the generation of the first cystatin C–deficient mouse to test its function in cancers. When melanoma cells were injected intravenously, cystatin C–deficient lungs showed reduced metastasis, but subcutaneous growth of melanoma cells was not different from that in control mice [23]. In contrast, cystatin C overexpression was associated with decreased glioblastoma cell invasion *in vitro* and tumor growth *in vivo* [24]. Such overexpression caused higher apoptosis *in vivo* for metastatic melanoma cells [25], and dramatically blocked lung metastasis of human fibrosarcoma cells (∼90%) in mice [26]. To date, however, there has been no rigorous experimental examination of how this cathepsin inhibitor participates in tumor growth.

In this study, we introduced cystatin C mutant alleles into the invasive epidermis multistage squamous-cell carcinoma (SCC) mouse model [27], in which the human keratin-14 promoter/enhancer drove the expression of human papillomavirus HPV16 early region genes, including the E6/E7 oncogenes, to test whether absence of this dominant cathepsin inhibitor alters the progression of premalignant dysplasia. This established dysplasia to carcinoma model [27] is characterized by temporally and histopathologically reproducible, multi-stage progression of dysplasia by 3 months to SCC by 9 months. Accordingly, it is ideal for evaluation of the effect of cystatin C on cancer progression via the gene knockout approach.

## Materials and Methods

### Mice and ethics

Cystatin C–deficient (*Cstc^−/−^*) mice (C57BL/6/S129 background) [23] were backcrossed to an FVB/n background (Jackson Laboratories, Bar Harbor, ME) for >5 generations. These mice were crossbred with K14-HPV16 transgenic mice [27] (kindly provided by Dr. Lisa Coussen from the University of California, San Francisco), in the same FVB/n genetic background to ensure early-stage (at 3 months) dysplasia. K14-HPV16 transgenic *Cstc^−/−^* and *Cstc^+/+^* wild-type littermates were sacrificed at 3 months of age. Chest and ear skin were collected and immediately embedded in compound OCT (optimum cutting temperature) for frozen section preparation (6 µm) or pulverized in liquid nitrogen to prepare tissue extracts for protein (lysed in a pH 5.5 buffer containing 1% Triton X-100, 40 mM sodium acetate, and 1 mM EDTA) or total RNA (lysed in TRIzol reagent; Stratagene, La Jolla, CA) preparation.

To test CatS and cystatin C expression during the progression of hyperplasia and dysplasia, we harvested skin tissues from K14-HPV16 transgenic mice at 1 month, 3 months, and 6 months of age for immunoblot analysis. Due to severe skin premalignant dysplasia from the K14-HPV16 transgenic *Cstc^−/−^* mice after 3 months of age, we were unable to study mice beyond this time point, for compliance with the humane standard of the Animal Research Committee of Harvard Medical School (approved animal protocol number 03759).

### Real-time PCR

Real-time polymerase chain reaction (RT-PCR) was used to determine cathepsin transcript levels, including CatK, CatL, CatS, and CatB, in both chest skin and ear skin from 3-month-old K14-HPV16 transgenic *Cstc^+/+^* and *Cstc^−/−^* mice. Total cellular RNA was extracted from TRIzol reagent-treated tumor tissue extracts. RNase-free DNase (Ambion, Austin, TX) was used to remove genomic DNA contaminants. Equal amounts of RNA were reverse-transcribed, and quantitative PCR was assessed in a single-color RT-PCR detection system (Stratagene). The level of each cathepsin transcript was normalized to that of the β-actin transcript.

### Western blot

Protein concentration of each tumor extract in pH 5.5 buffer was determined using Bio-Rad DC protein assay kit (Bio-Rad, Hercules, CA). An equal amount of protein (30 µg/sample) of each tissue extract preparation was separated on 14% SDS-PAGE, followed by immunoblot analysis with rabbit anti-human cathepsins S, L, and K polyclonal antibodies (1∶1000, Calbiochem, San Diego, CA) or rabbit anti-human cystatin C polyclonal antibodies (1∶1000; DAKO Corp., Carpinteria, CA), all of which cross-reacted with mouse gene products. Goat anti-mouse β-actin polyclonal antibodies (1∶5000, Santa Cruz Biotechnology, CA) were used to determine equal protein loading. Densitometry analysis (ImageJ software, Bethesda, MD) was used to determine the relative cathepsin protein levels in skin and ear tissues from K14-HPV16 transgenic *Cstc^+/+^* and *Cstc^−/−^* mice.

### ELISA

To determine systemic cathepsin levels, mouse serum samples from *Cstc^+/+^* and *Cstc^−/−^* mice were used directly for ELISA. ELISA for CatS, matrix metalloproteinase-9 (MMP-9) (R&D Systems, Inc., Minneapolis, MN), and CatL (Bender MedSystems Inc., Burlingame, CA) were used according to the manufacturers' protocols.

### Collagenase and elastase activity assay

A total of 20 µg of chest and ear skin extract from K14-HPV16 transgenic *Cstc^+/+^* and *Cstc^−/−^* mice was combined to a final volume of 100 µl in EDTA-containing pH 5.5 buffer that was optimized for measuring cysteinyl cathepsin activities, but not MMPs or other neutral serine proteases. Fluorogenic elastin (10 µg/well, DQ elastin, Invitrogen, Carlsbad, CA) or type I collagen (10 µg/well, Calbiochem) were added to a 96-well plate. After adding 3 mM of dithiothreitol (Sigma, St. Louis, MO), the plate was incubated at 37°C for 3 days and read at an excitation of 505 nm and emission of 515 nm. Data were presented as relative fluorescent units.

### Immunohistology

Frozen sections (6 µm) prepared from chest and ear skin of the K14-HPV16 transgenic *Cstc^+/+^* and *Cstc^−/−^* mice were used for conventional histological (hematoxylin and eosin stain) and immunohistological analysis with rabbit anti-human CatS polyclonal antibody (1∶170, cross-reacts with mouse CatS) [28]; rabbit anti-human Ki67 polyclonal antibody (1∶750, NovoCastra Laboratories Ltd, Newcastle, UK); rat anti-mouse CD31 (1∶150), Mac-3 (1∶900), CD4 (1∶90), and CD8 (1∶100) monoclonal antibodies (Pharmingen, San Diego, CA); rabbit anti-mouse laminin 5 fragment γ2 polyclonal antibody (1∶250) [29]; and the TUNEL staining kit (ApopTag® Plus Peroxidase *In Situ* Apoptosis Kit, Millipore, Billerica, MA). Immunostaining was analyzed using computer-assisted image analysis software (Image-Pro Plus; Media Cybernetics, Bethesda, MD). Data were presented as antigen-positive area (%) or cell number per mm^2^.

### Statistical analysis

Due to our relatively small sample sizes and abnormal data point distribution, we used the non-parametric Mann-Whitney *U* test to examine the statistical significance for all data in this study. All data were presented as mean ± SEM. *P*<0.05 was considered statistically significant.

## Results

### Cystatin C and CatS expression in early hyperplasia and dysplasia stages from K14-HPV16 transgenic mice

**Figure 1:**
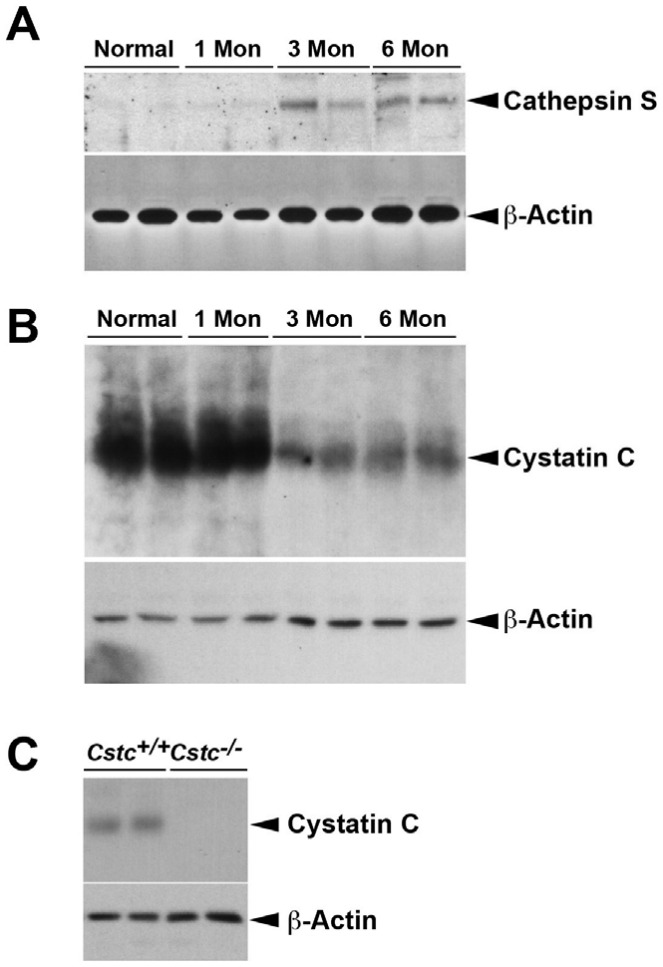
Immunoblot analysis for CatS and cystatin C in mouse chest skin tissue extracts. Chest skin tissue extracts from K14-HPV transgenic *Cstc^+/+^* mice at different stages (1 month, 3 months, and 6 months) were analyzed for CatS (A) and cystatin C (B) expression. Cystatin C immunoblot analysis confirmed the absence of cystatin C in K14-HPV transgenic *Cstc^−/−^* mice in chest skin tissue extracts from 3-month-old K14-HPV transgenic *Cstc^+/+^* and *Cstc^−/−^* mice (C). Equal protein loading (30 µg/lane) was confirmed with a β-actin immunoblot.

As discussed, expression of cathepsins and their inhibitors varies at different stages of tumor growth [22]. To examine whether CatS and cystatin C were also expressed differently during the progression of premalignant dysplasia, we collected chest skin tumor tissues at different stages from K14-HPV16 transgenic mice that were wild-type for cystatin C, including those tumors established at early (3 months) and late (6 months) stages of dysplasia. Immunoblot analysis demonstrated that CatS protein levels plateaued at 3∼6 months of age. Normal skins or those from the early stage (e.g., 1 month) had negligible CatS proteins (Figure 1A). In contrast, normal skins had high levels of cystatin C protein, which then decreased during premalignant progression, although cystatin C protein did not completely disappear at the 3-month and 6-month progression stages (Figure 1B).

### Cystatin C deficiency enhanced SCC in K14-HPV16 transgenic mice

**Figure 2:**
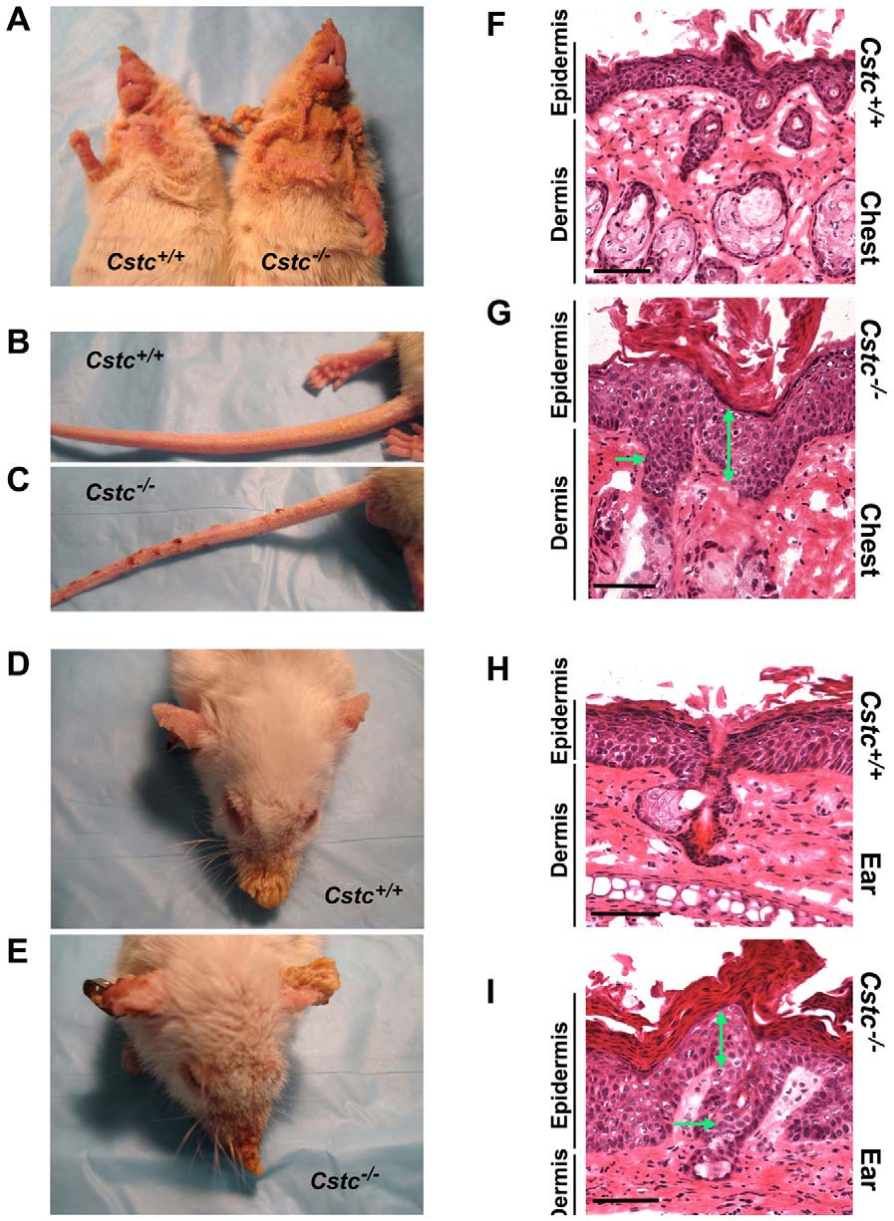
Enhanced epidermal dysplasia in cystatin C-deficient K14-HPV16 transgenic mice. Premalignant dysplasia on 3-month-old K14-HPV16 transgenic *Cstc^+/+^* and *Cstc^−/−^* mouse chest skin (A), tail (B, C), face, and ear (D, E). Mouse genotypes are indicated. Histopathology of lesions from *Cstc^+/+^* and *Cstc^−/−^* mice (F, H, and G, I, respectively) taken from chest skin (F, G) and ear skin tissue (H, I), showing partial-thickness atypia of epidermal cells in *Cstc^+/+^* mice, and full-thickness atypia (double headed arrow, SCC *in situ*) in *Cstc^−/−^* mice. Both dermis and epidermis are indicated. Also note that SCC in *Cstc^−/−^* mice extends to involve the adnexal epithelium of hair follicles (horizontal arrow).

To test the effect of cystatin C in SCC, we introduced the K14-HPV16 transgene into *Cstc^−/−^* and *Cstc^+/+^* littermate control mice by crossbreeding *Cstc^−/−^* mice [23] with K14-HPV16 transgenic mice [27]. Genomic DNA PCR confirmed the cystatin C genotypes in K14-HPV16 transgenic mice (data not shown). Due to the extent of malignant dysplasia in *Cstc^−/−^* K14-HPV16 transgenic mice at later time points (after 3 months), for humane purposes, we sacrificed the K14-HPV16 transgenic mice that were *Cstc^+/+^*and *Cstc^−/−^* at 3 months of age for the remainder of the study. Anti-human cystatin C polyclonal antibody–mediated immunoblot analysis of chest skin tissue extracts from 3-month-old *Cstc^−/−^* and *Cstc^+/+^* K14-HPV16 transgenic mice confirmed the absence of cystatin C in *Cstc^−/−^* mice (Figure 1C). At the 3-month time point, we observed SCC formation that was clinically more pronounced in skin from the chest (Figure 2A), tail (Figure 2B/C), face, and ear (Figure 2D/E) from the *Cstc^−/−^* mice as compared with that of the *Cstc^+/+^* mice. These differences were confirmed histologically (Figure 2F–I); whereas back and ear skin from *Cstc^+/+^* mice exhibited dysplasia (nuclear atypia and disordered maturation in epidermal cells, primarily within the lowermost layers), abnormalities were present uniformly throughout a thickened and more hyperkeratotic epidermal layer in the *Cstc^−/−^* mice, thus qualifying for a designation of SCC *in situ*. Such SCC formation or dysplasia was absent in *Cstc^−/−^* or *Cstc^+/+^* mice without the K14-HPV16 transgene (not shown). Therefore, absence of cystatin C enhanced dysplasia to SCC progression in site-matched skin in the K14-HPV16 transgenic mice.

### Cystatin C deficiency increased cysteinyl cathepsin expression and activities

**Figure 3:**
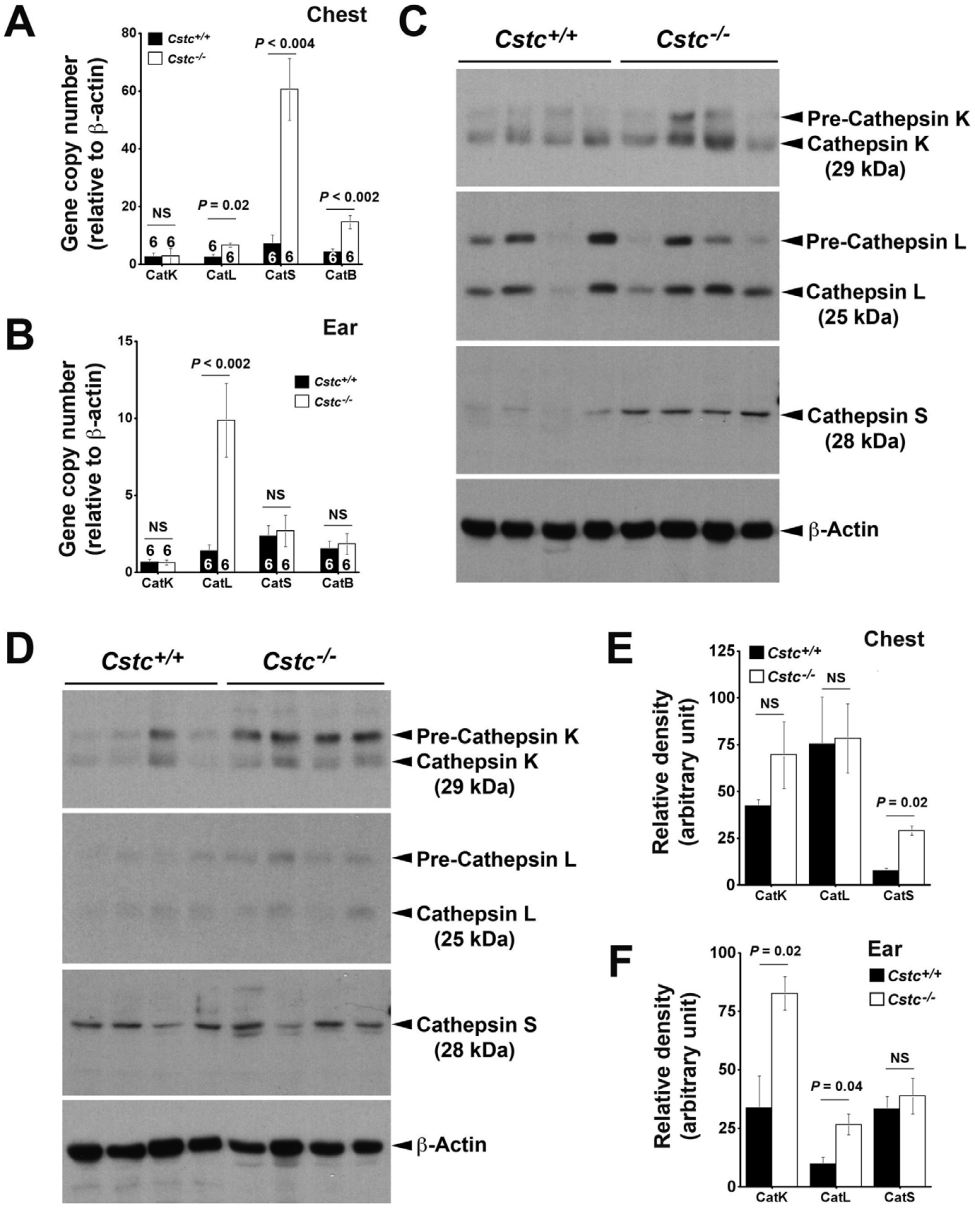
Cystatin C deficiency affects cathepsin expression and activity. A/B: K14-HPV16 transgenic *Cstc^+/+^* and *Cstc^−/−^* mouse chest (A) and ear (B) skin tissue real-time PCR analysis for cathepsins K, L, S, and B. Number of mice per group is indicated in each bar. C/D: K14-HPV16 transgenic *Cstc^+/+^* and *Cstc^−/−^* mouse chest (A) and ear (B) skin tissue extract immunoblot analysis for cathepsins K, L, and S. Actin immunoblots were used for protein loading controls. E/F: Densitometric analysis for the cathepsin immunoblot signals from the chest (E) and ear (F) shown in C and D. Data are mean ± SEM. p<0.05 was considered statistically significant, non-parametric Mann-Whitney *U* test. NS: no significant difference.

**Figure 4 pone-0013973-g004:**
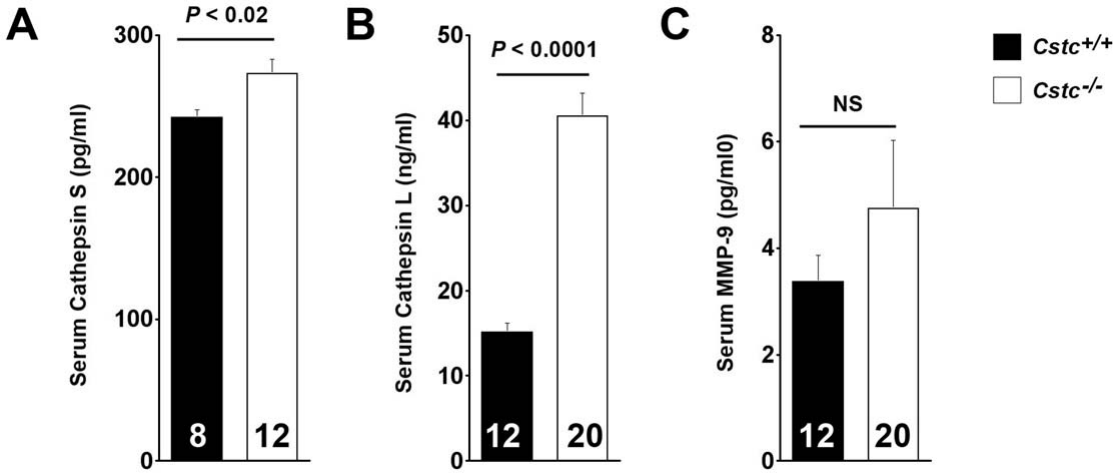
Serum protease levels. Serum levels of CatS (A), CatL (B), and MMP-9 (C) in K14-HPV16 transgenic *Cstc^+/+^* and *Cstc^−/−^* mice. Number of mice per experimental group is indicated in each bar. Data are mean ± SEM. NS: no significant difference.

**Figure 5 pone-0013973-g005:**
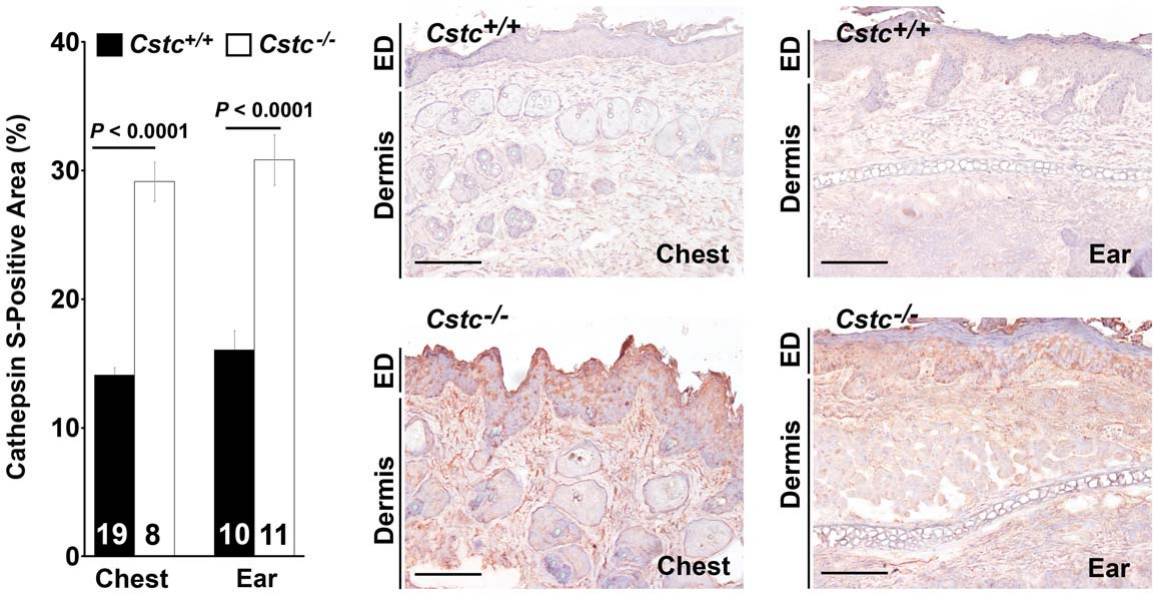
Immunostaining for CatS in chest and ear skin tumor sections. Number of mice per experimental group is indicated in each bar. Representative sections are shown to the right. Both dermis and epidermis (ED) are indicated. Data are mean ± SEM. Data are presented as % of CatS-positive area. Scale bar: 100 µm.

Enhanced SCC formation in *Cstc^−/−^* K14-HPV16 transgenic mice compared with those wild-type for cystatin C suggests the importance of this endogenous cathepsin inhibitor in early tumor progression *in vivo*. The development of SCC in *Cstc^−/−^* K14-HPV16 transgenic mice possibly results from altered counterbalance between cystatin C and cathepsins. To test this hypothesis, we prepared total RNA from chest and ear skin and performed RT-PCR. Absence of cystatin C significantly increased the RNA levels of cathepsins L, S, and B in the chest skin and of CatL in the ear skin (Figure 3A/B). Tumor tissue extract immunoblot analysis using protein samples from the chest skin (Figure 3C) or the ear skin (Figure 3D) also revealed significantly increased CatS protein in the chest skin extracts (Figure 3E) and CatK and CatL proteins in the ear skin extracts (Figure 3F), as determined by densitometric measurements. In serum from K14-HPV16 transgenic *Cstc^+/+^* and *Cstc^−/−^* mice, we made similar observations. Serum from the *Cstc^−/−^* mice had significantly higher levels of both CatS (Figure 4A) and CatL (Figure 4B) than that from the *Cstc^+/+^* mice, whereas cystatin C deficiency did not significantly affect serum MMP-9 levels (Figure 4C). Chest and ear skin immunohistological analysis also revealed increased expression of CatS throughout the epidermis and dermis from *Cstc^−/−^* mice, compared with those from *Cstc^+/+^* mice (Figure 5).

**Figure 6:**
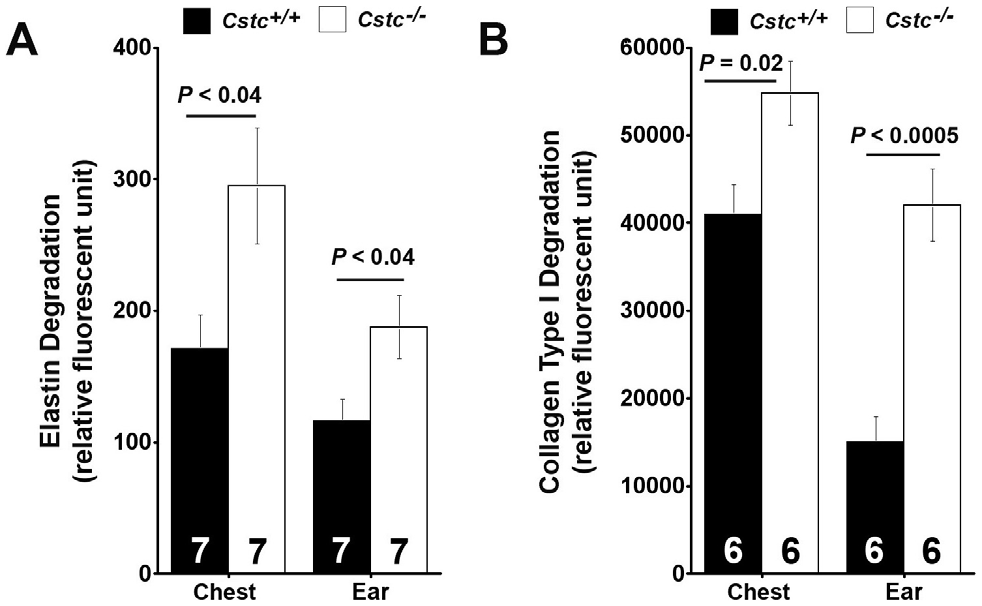
Mouse chest and ear skin tissue extract elastase and collagenase assays. K14-HPV16 transgenic *Cstc^−/−^* mouse chest and ear skin tissue extracts showed significantly increased elastase (A) and collagenase (B) activities compared those of the *Cstc^+/+^* mice. Number of mice per group is indicated in each bar. Data are mean ± SEM.

Absence of cystatin C also enhanced tumor tissue extract cathepsin activities. Upon incubating chest and ear skin extracts in pH 5.5 buffer, which measures only cysteinyl cathepsins [30], with fluorogenic elastin and collagen, two common matrix protein substrates for cathepsins S, L, K, and B, we found that tumor tissue extracts from both the chest skin and ear skin from *Cstc^−/−^* mice had significantly higher elastase (Figure 6A) and collagenase (Figure 6B) activities than those from *Cstc^+/+^* mice. Together, our data demonstrated increased expression and activities of cysteinyl cathepsins in the absence of cystatin C.

**Figure 7:**
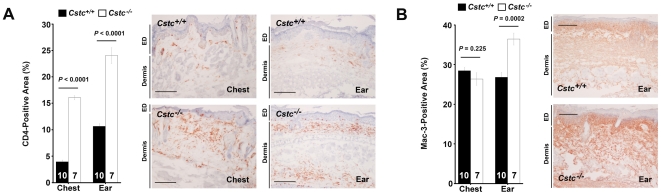
Immunostaining for inflammatory cells in chest and ear skin tumor sections. CD4^+^ T cells (**A**) and Mac-3^+^ macrophages (**B**) in K14-HPV16 transgenic *Cstc^+/+^* and *Cstc^−/−^* mouse chest and ear skin tissue sections. Number of mice per group is indicated in each bar. Representative sections are shown to the right. Both dermis and epidermis (ED) are indicated. Data are mean ± SEM. Scale bar: 100 µm.

Tumor progression often accompanies inflammatory cell infiltrations [31]–[33], including monocytes/macrophages, B and T lymphocytes, NK cells, and neutrophils. These infiltrates often influence the tumor progression directly. For example, in the same K14-HPV16 transgenic mice used in this study, deficiency of CD4^+^ T cells delays neoplastic progression and lowers tumor incidence [33]. These cells are rich in cathepsin expression and activities [34], [35]. Therefore, increased cysteinyl cathepsin expression and activities in SCC chest and ear skin tissues from *Cstc^−/−^* mice can be the results of the absence of cystatin C as well as of enhanced inflammatory cell infiltration. To examine this hypothesis, we immunostained both the chest and ear sections from *Cstc^+/+^* and *Cstc^−/−^* mice for CD4, CD8, and macrophage marker Mac-3. Both chest and ear sections from *Cstc^−/−^* mice contained significantly higher percentages of CD4-positive areas than did the *Cstc^+/+^* mice (Figure 7A). Mac-3–positive areas were also higher in the ear sections, but not in the chest sections, from *Cstc^−/−^* mice than from *Cstc^+/+^* mice (Figure 7B). There were no differences, however, in CD8-positive areas in chest sections (10.53±4.35 *vs.* 8.40±3.44, *P* = 0.252) and ear sections (6.37±3.79 *vs.* 7.34±0.56, *P* = 0.096) between *Cstc^−/−^* and *Cstc^+/+^* mice. Increased infiltration of CD4^+^ T cells and Mac-3^+^ macrophages in *Cstc^−/−^* mice may account in part for enhanced lesion cathepsin activities in chest and ear skin from these mice (Figures 3C–3F, 5, and 6).

### Decreased apoptosis and increased cell proliferation in cystatin C–deficient SCC

**Figure 8:**
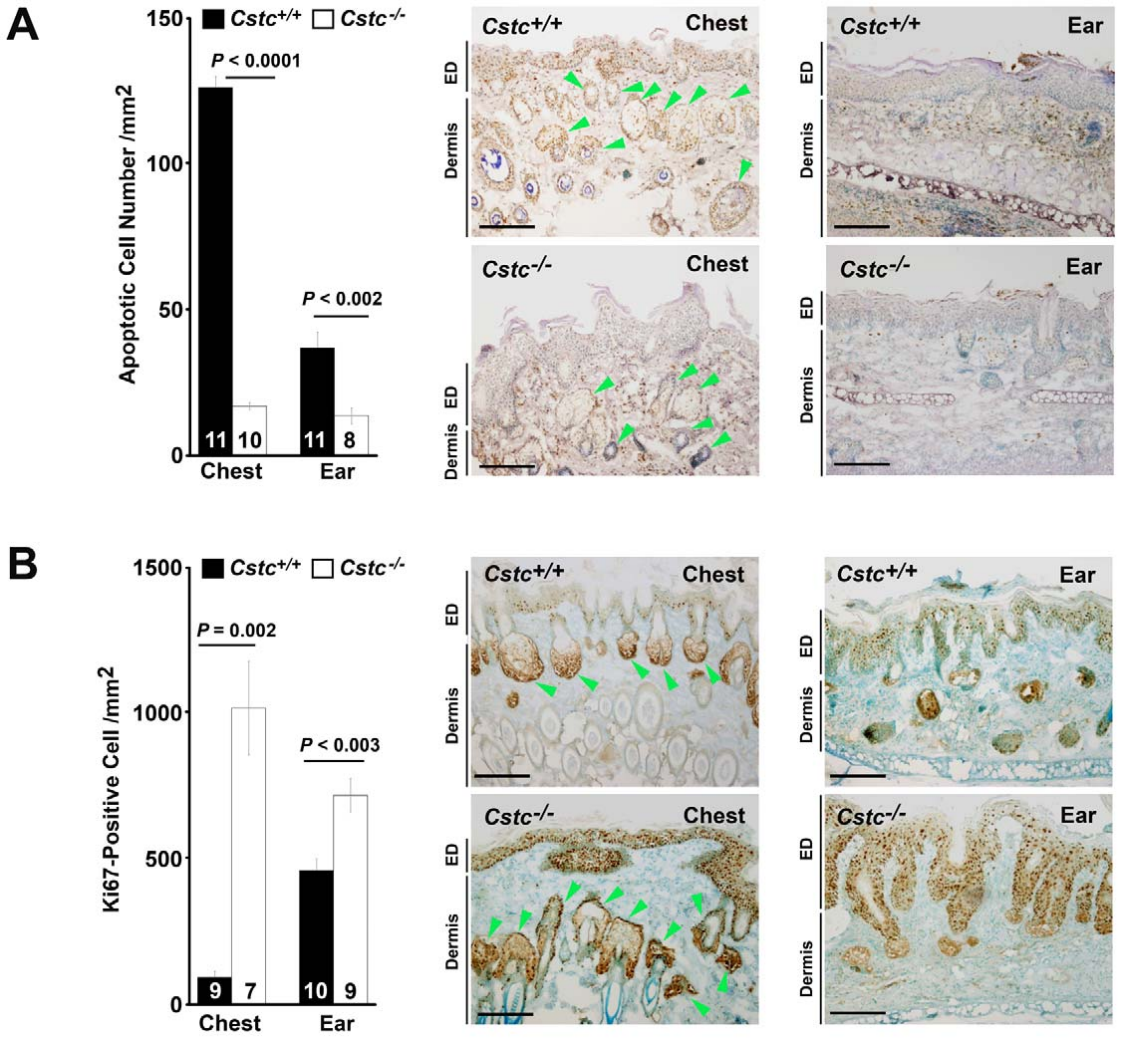
Immunostaining for apoptotic and proliferating cells in chest and ear skin tumor sections. K14-HPV16 transgenic *Cstc^+/+^* and *Cstc^−/−^* mouse chest and ear skin tissue section immunostaining for TUNEL (A) and Ki67 (B). Representative sections are shown in the right panels. Dermis and epidermis (ED) are indicated. Arrowheads indicate hair sebaceous units. Data are mean ± SEM. Scale bar: 100 µm.

Tumor cell apoptosis and proliferation are major determinants of growth or progression of cancers [36], [37]. The development of fully evolved *in situ* SCC in *Cstc^−/−^* mice, but not in *Cstc^+/+^* mice, suggests that cystatin C deficiency may reduce tumor cell apoptosis and/or increase tumor cell proliferation. To test this possibility, we immunostained both the chest and ear skin tissue frozen sections for TUNEL and for the cell proliferation marker Ki67. Consistent with our hypothesis, tumor tissues (chest and ear skin) from *Cstc* ^−/−^ mice had significantly fewer total apoptotic cells than those from *Cstc^+/+^* mice (Figure 8A). In *Cstc^+/+^* mouse chest skin, most apoptotic cells were noted in the epidermis and hair sebaceous unit areas. Relatively fewer apoptotic cells appeared in the dermis. However, in *Cstc^−/−^* mouse chest skin, both the epidermis and sebaceous unit showed noteworthy reduction of apoptotic cells. In contrast, apoptotic cells from the *Cstc^+/+^* mouse ear skin clustered in the dermis, while ear skin from the *Cstc^−/−^* mice had significantly reduced levels of such apoptotic cells (Figure 8A). Significantly more Ki67-positive proliferating cells were identified in the epidermis and sebaceous units from chest skin of the *Cstc^−/−^* mice than in those from the *Cstc^+/+^* mice (Figure 8B). Reduced apoptosis, but increased proliferation in the epidermis and sebaceous unit of *Cstc^−/−^* mice, thus correlates with progression to SCC *in situ*. Similar findings were documented in the *Cstc^−/−^* mouse ear skin. In the epidermis of *Cstc^−/−^* mouse ear skin, we detected significantly more Ki67-positive cells than in that of the *Cstc^+/+^* mice (Figure 8B). In summary, cystatin C deficiency increased cell proliferation but decreased cell apoptosis, thus correlating with progression to SCC *in situ* in both chest and ear skin, events that likely also developed at other similarly affected sites (e.g., tail and face skin; Figure 2).

### Cystatin C deficiency led to increased angiogenesis

**Figure 9:**
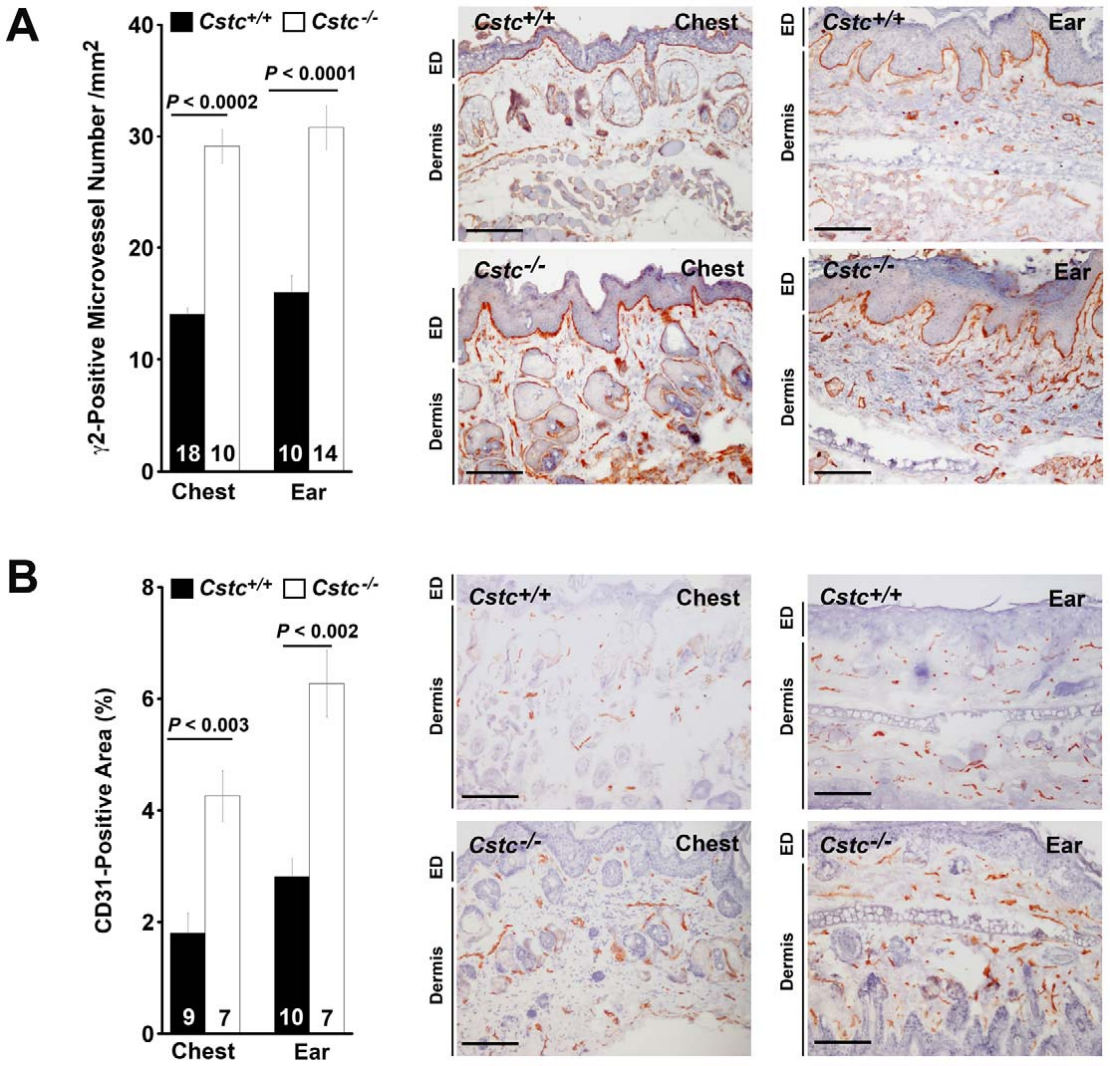
Immunostaining for microvessels in chest and ear skin tumor sections. K14-HPV16 transgenic *Cstc^+/+^* and *Cstc^−/−^* mouse chest and ear skin tissue section immunostaining for laminin-5 fragment γ2 (A) and CD31 (B). Number of mice per experimental group is indicated in each bar. Representative sections are shown in the right panels. Dermis and epidermis (ED) are indicated. Data are mean ± SEM. Scale bar: 100 µm.

Cysteinyl cathepsin-mediated angiogenesis is important in cancer progression and tumor growth. We reported that in the pancreatic islet cell carcinoma RIP-Tag2 model, absence of CatS significantly reduced tumor growth [13]. Mechanistically, we demonstrated that CatS mediates extracellular matrix laminin-5 degradation, followed by the production of a pro-angiogenic peptide from laminin-5 proteolysis, named γ2 [13], [29]. In CatS-deficient mice, along with reduced tumor growth, we detected significant reduction of this γ2 fragment, and concomitantly, greatly impaired angiogenesis [13]. Along with producing the γ2 fragment from laminin-5, cysteinyl cathepsins mediate other matrix protein degradation, all of which relate to angiogenesis and tumor growth. In the present study, we conjectured that the absence of cystatin C caused increased cathepsin expression and activities (Figures 3–6) and enhanced pro-angiogenic γ2 production with promotion of angiogenesis, leading to increased progression to SCC (Figure 2). To test this hypothesis, we performed immunohistological analysis of both chest and ear skin from the *Cstc^+/+^* and *Cstc^−/−^* mice. Consistent with our hypothesis, we detected significantly more γ2-positive microvessels in the dermis of both chest and ear skin of *Cstc^−/−^* mice than in those of *Cstc^+/+^* mice (Figure 9A). Along with increased γ2 production in *Cstc^−/−^* mouse chest and ear skin, we observed significant enhancement of CD31-positive microvessels in the skin of these mice, more than twice as many as those from *Cstc^+/+^* mice (Figure 9B). Increased angiogenesis in *Cstc^−/−^* mouse skin may relate to associated progression to SCC.

## Discussion

Protease and protease inhibitor counterbalance prove physiologically important and pathologically relevant in human diseases. Increased protease activities, either by increased protease expression or decreased protease inhibitor expression, lead to many common human disorders, such as cardiovascular diseases, metabolic conditions, bone metabolic dysfunction, cancers, and neurological complications. We have previously shown that the absence of cystatin C enhanced atherogenesis in apolipoprotein E-deficient (*Apoe^−/−^*) mice [38]. One mechanism of advanced atherogenesis in these *Apoe^−/−^* mice, increased cathepsin activities, leads to enhanced elastinolysis and collagenolysis, and thus to blood-borne leukocyte accumulation in the neointima. In the present study, although using a different disease model, we found that cystatin C deficiency results in enhanced SCC progression involving a similar mechanism to what we have observed in the cardiovascular system. Absence of this cathepsin inhibitor enhanced cathepsin expression and activity, which altered epidermal cell kinetics and advanced angiogenesis, thereby expediting cancer progression.

The function of cathepsins in angiogenesis has been well studied in several disease models [39]. In general, angiogenesis involves extensive extracellular matrix protein degradation and tissue remodeling, which requires protease involvement. But cathepsins can achieve more than just matrix protein degradation. *In vitro* studies showed that CatB and CatL can liberate collagen IV, laminin, and fibronectin from the basement membrane, generate gelatinolytic fragments, and initiate a “metastatic cascade,” thereby promoting angiogenesis and tumor invasion [40]. We recently have shown that CatS degrades the anti-angiogenic peptides arresten, canstatin, and tumstatin, all of which constitute collagen IV degradation products [13], [41]. Further, CatS degrades the natural basement membrane matrix protein laminin-5, and generates pro-angiogenic γ2 fragments *in vitro* [13], [29]. Cysteinyl cathepsin proteolysis of these latent matrix proteins therefore is critical in regulating angiogenesis, and thus in tumor invasion, as we found in the CatS-deficient RIP-Tag2 model. Absence of CatS reduced γ2 fragment production, and consequently reduced islet-cell carcinoma growth [13]. In the present study, we also detected significantly higher amounts of CatS (Figure 5) and CatS-derived laminin-5 γ2 fragments (Figure 9A) in chest and ear skin lesions from *Cstc^−/−^* mice than in those from *Cstc^+/+^* mice. These observations furnish a mechanistic explanation of the increased angiogenesis detected from the chest and ear skin of the *Cstc^−/−^* mice, compared with those from the *Cstc^+/+^* mice (Figure 9B).

Cancer cell proliferation and apoptosis closely relate to angiogenesis, and therefore are important in tumor growth. Angiogenesis provides cancer cells with sufficient nutrients for survival and proliferation; reduced angiogenesis thus may cause cancer cell apoptosis or decreased cell proliferation. In return, cancer-cell apoptosis and proliferation can also regulate angiogenesis. Cysteinyl cathepsins can participate importantly in these processes. When co-cultured with human glioblastoma cells, human microvascular EC increased the invasiveness of the glioblastoma cells. On the other hand, glioblastoma cells stimulated microvascular EC proliferation, which CatB-selective inhibitor CA074me can impair [42]. Therefore, increased expression of CatB may enhance EC proliferation i*n vivo*. Although we saw increased expression of several important cathepsins and enhanced cancer cell proliferation in chest and ear skin sections from *Cstc^−/−^* mice (Figure 8B), whether the increase of proliferation associated with angiogenesis remains unknown. Our observation of reduced cancer-cell apoptosis in chest and ear skin from *Cstc^−/−^* mice (Figure 8A) gives some support to this hypothesis.

Although we anticipated enhanced angiogenesis and tumor growth in *Cstc^−/−^* mice, several aspects of our results remain unexplained, and more studies are required for further understanding of the role of cathepsins in tumorigenesis. First, we frequently have seen that absence of one protease affects the expression of the others — for example, vascular cells from serine protease chymase-deficient [43] or tryptase-deficient (unpublished data) mice express significantly reduced levels of cathepsins. We demonstrated in this study that deficiency of cystatin C increased both RNA and protein levels of most, if not all, tested cathepsins. Second, cathepsins are thought to promote cell apoptosis — for example, CatB cleaves the anti-apoptotic protein Bcl-2 member Bid and creates a pro-apoptotic signal of mitochondrial cytochrome C release [44]. Osteosarcoma cell line U2OS, overexpressing HPV oncogene E7 and cell cycle regression regulator p21, undergoes apoptosis in a CatB-dependent but caspase-independent manner. CatB-selective inhibitor CA074me significantly blocked U2OS cell death [45]. Thus, CatB-deficient mice showed reduced cerebral cell losses, whereas a cystatin mutation resulted in increased apoptosis of cerebellar granule cells [46]. A direct role for cathepsins in promoting apoptosis does not explain our finding in this study of increased cathepsin expression and activities, but reduced cancer apoptosis (Figures 3–6, Figure 8A). One possibility is that increased cathepsin expression and activities in *Cstc^−/−^* mice enhanced extracellular matrix processing, which may explain why we detected more laminin-5 fragment γ2 (Figure 9A) and increased angiogenesis (Figure 9B). Compared with the chest and ear skin from *Cstc^−/−^* mice, we found significantly lower numbers of microvessels in the *Cstc^+/+^* mice (Figure 9B). Reduced angiogenesis in *Cstc^+/+^* mice may affect nutrient supply, thus increasing cell death and reducing proliferation, although a detailed mechanism of cathepsin function in this SCC cancer model requires further investigation. Third, it is conceivable that angiogenesis is essential for tumorigenesis after angiogenic switch, but less so in early stages. Our data demonstrated that enhanced cathepsin expression expedited malignant transformation, which also can be caused by alterations in growth factor levels, inflammatory cell infiltration (Figure 7), and associated intracellular signaling. Mechanisms in addition to enhanced angiogenesis may account for more advanced tumorigenesis in *Cstc^−/−^* mice than in *Cstc^+/+^* mice.

In conclusion, this study provides additional *in vivo* evidence to support direct participation of cysteinyl cathepsins in tumor growth. Based on these findings, we conjecture that the absence of cystatin C also may increase the progression and growth of other types of tumors. Thus, inhibition of cysteinyl cathepsins, either by small molecular inhibitors [47], antibody therapy [48], or retrovirus-mediated cathepsin inhibitor delivery [49], holds potential for the development of novel cancer therapies.
